# Implementing a vector surveillance-response system for chagas disease control: a 4-year field trial in Nicaragua

**DOI:** 10.1186/s40249-016-0225-7

**Published:** 2017-03-06

**Authors:** Kota Yoshioka, Doribel Tercero, Byron Pérez, Jiro Nakamura, Lenin Pérez

**Affiliations:** 1 Doctor of Public Health Program, Harvard T.H. Chan School of Public Health, Boston, USA; 2 School of Tropical Medicine and Global Health, Nagasaki University, Nagasaki, Japan; 3 Former Chagas Disease Control Project, Japan International Cooperation Agency, Managua, Nicaragua; 4 Department of Disease Prevention, Ministry of Health in Nicaragua, Managua, Nicaragua

**Keywords:** Chagas disease, *Triatoma dimidiata*, Vector control, Surveillance-response system, Programme integration, Nicaragua

## Abstract

**Background:**

Chagas disease is one of the neglected tropical diseases (NTDs). International goals for its control involve elimination of vector-borne transmission. Central American countries face challenges in establishing sustainable vector control programmes, since the main vector, *Triatoma dimidiata*, cannot be eliminated. In 2012, the Ministry of Health in Nicaragua started a field test of a vector surveillance-response system to control domestic vector infestation. This paper reports the main findings from this pilot study.

**Methods:**

This study was carried out from 2012 to 2015 in the Municipality of Totogalpa. The Japan International Cooperation Agency provided technical cooperation in designing and monitoring the surveillance-response system until 2014. This system involved 1) vector reports by householders to health facilities, 2) data analysis and planning of responses at the municipal health centre and 3) house visits or insecticide spraying by health personnel as a response. We registered all vector reports and responses in a digital database. The collected data were used to describe and analyse the system performance in terms of amount of vector reports as well as rates and timeliness of responses.

**Results:**

During the study period, *T. dimidiata* was reported 396 times. Spatiotemporal analysis identified some high-risk clusters. All houses reported to be infested were visited by health personnel in 2013 and this response rate dropped to 39% in 2015. Rates of insecticide spraying rose above 80% in 2013 but no spraying was carried out in the following 2 years. The timeliness of house visits improved significantly after the responsibility was transferred from a vector control technician to primary health care staff.

**Conclusions:**

We argue that the proposed vector surveillance-response system is workable within the resource-constrained health system in Nicaragua. Integration to the primary health care services was a key to improve the system performance. Continual efforts are necessary to keep adapting the surveillance-response system to the dynamic health systems. We also discuss that the goal of eliminating vector-borne transmission remains unachievable. This paper provides lessons not only for Chagas disease control in Central America, but also for control efforts for other NTDs that need a sustainable surveillance-response system to support elimination.

**Electronic supplementary material:**

The online version of this article (doi:10.1186/s40249-016-0225-7) contains supplementary material, which is available to authorized users.

## Multiligual abstracts

Please see Additional file 1 for translations of the abstract into the six official working languages of the United Nations.

## Background

Neglected tropical diseases (NTDs) are one of the most important challenges to address in reducing global health disparities. In 2012, the World Health Organization (WHO) released an ambitious roadmap to accelerate elimination of some NTDs [1]. Elimination is defined as the reduction to zero in the incidence of infection in a given geographical area, with continuous measures to prevent re-establishment of transmission [2]. By definition, elimination of NTDs requires implementation of a sustainable programme that can suppress the risk of re-infection for a long time. A concept of “surveillance and response” has therefore been proposed as a key sustainable strategy towards NTD elimination, particularly for resource-constrained countries [3].

Surveillance and response involves a sequence of activities: 1) definition of disease identification; 2) detection of pockets of active transmission; 3) analysis of collected data; and 4) implementation of response packages [4]. Compared to the classical public health surveillance, the surveillance and response is an innovative concept because it seeks to use a minimal essential set of data [5] and it involves implementation of responses [4]. In resource-constrained settings, the classical surveillance often results in ‘information overload’ and little or delayed feedback [5]. The surveillance and response seeks a balance between data management and responsive actions, which is crucial to suppress NTD transmission risk continuously.

Chagas disease is one of the NTDs that require sustainable control programmes. It is caused by a protozoan, *Trypanosoma cruzi*, which is usually transmitted to humans by triatomine bug vectors in Latin American counties [6]. Since the triatomine vectors usually infest poorly constructed dwellings, Chagas disease affects disproportionally the poor. Other means of transmission include congenital transmission, blood transfusion, organ transplants, laboratory-acquired contamination and oral transmission [7, 8]. As detection and treatment of patients used to be difficult and ineffective, the interruption of *T. cruzi* transmission through vectors and blood transfusion has been central to Chagas disease control [9]. Hitherto, many Latin American countries have implemented Chagas disease control programmes successfully and the estimated annual number of new cases from vector-borne transmission reduced from 41 200 in 2005 to 30 000 in 2010 [10, 11]. The WHO roadmap proposes the elimination of Chagas disease transmission in Latin America by 2020 [1].

Central American countries launched a regional initiative that shared the following objectives: 1) interruption of *T. cruzi* transmission by allochthonous vector *Rhodnius prolixus*; 2) reduction of *T. cruzi* transmission by autochthonous vector *Triatoma dimidiata*; and 3) elimination of *T. cruzi* transmission by blood transfusion [12]. For vector control, the countries adopted a traditional three-phase strategy that consists of 1) a preparatory phase, mapping vector distribution and programming resources, 2) an attack phase, including large-scale insecticide spraying of houses, and 3) a surveillance phase for the detection of residual vectors [9]. This vector control strategy was proven to be effective when *T. cruzi* transmission by *Triatoma infestans* was successfully interrupted in some South American countries. In Central America, the same strategy nearly eliminated *R. prolixus*, which led to the rapid reduction of Chagas disease incidence in the region [13].

Field experiences against *T. dimidiata* in Central America show that the attack phase could reduce also its house infestation level significantly, however, this species can infest human dwellings repeatedly even after the initial spraying [14–17]. Frequent re-infestation by *T. dimidiata* is due to the vector’s capacity to adapt to a wide range of habitats, from indoor to sylvatic conditions, and to move among houses and villages [18, 19]. Because of *T. dimidiata* re-infestation, acute cases of Chagas disease have been reported even in previously-sprayed areas in El Salvador [20, 21]. The current challenge to eliminating Chagas disease transmission in Central America is how to prevent re-establishment of vector-borne transmission by *T. dimidiata*. As the traditional vector control strategy does not presuppose such frequent vector re-infestation, alternative control strategies are required [22], particularly in Central America.

Since *T. dimidiata* cannot be eliminated in Central America, a vector surveillance-response system must be sustained to minimize infection risks over time [22]. Such a surveillance-response system needs to be workable even in resource-constrained settings to succeed in Central America. Community participation, where community residents notify health programmes when they find vectors in their houses, is proposed as a key element [23]. Vector collection by householders is proven to be cost-effective and sustainable compared with manual searches by government inspectors [24, 25]. Several models of the community-based vector surveillance for Chagas disease control have been implemented in neighbour countries of Guatemala, El Salvador and Honduras [26, 27]. A workable vector surveillance-response system in Nicaragua, however, had not been developed.

The Nicaraguan Ministry of Health (MoH) therefore set up a small-scale field study to develop and test an entomological surveillance-response system for Chagas disease control that would work locally, drawing on technical support from the Japan International Cooperation Agency (JICA). In this paper, we describe the process of implementing a vector surveillance-response system and summarize the main results from a 4-year pilot study in Nicaragua. Further challenges and lessons from field experiences will be discussed.

## Methods

### Site

**Fig. 1:**
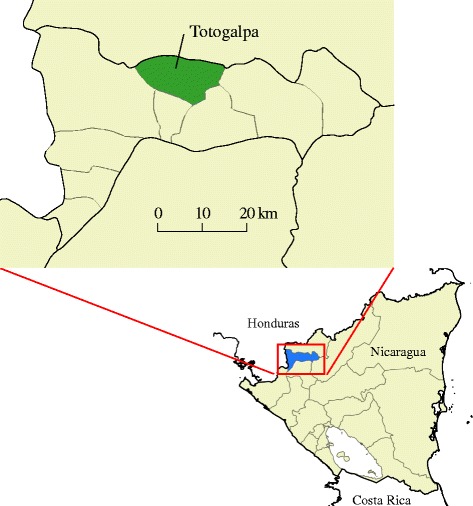
Location of the municipality of Totogalpa, Department of Madriz, Nicaragua

The municipality of Totogalpa, department of Madriz, was selected as a pilot site for developing the entomological surveillance-response system for Chagas disease control (Fig. 1). This municipality was used because it had reported the highest number of *T. cruzi*-infected cases in the country. The municipality of Totogalpa has a geographical area of 130 km^2^, with a population of approximately 13 000 spread across 39 communities.

### Local health system

In Nicaragua, the local health system is organized in a decentralized manner, under a national Family and Community Health Model, locally known as MOSAFC, the Spanish abbreviation. In line with the PAHO/WHO’s recommendations to renew primary health care (PHC) in the Americas [28], MOSAFC was established to improve access to and quality of health services [29].

In Totogalpa, one health centre in the municipal capital has responsibility for managing all governmental health programmes. This health centre is managed by a municipal health director and has a vector control technician (VCT) in charge of vector control programmes. The municipality is divided into five sectors (Table 1). Each sector has a PHC team, usually a physician, nurse and auxiliary nurse, to provide general health services to the population. These PHC teams work closely with community health volunteers, who are community residents and support the PHC teams’ work without payment.

**Table 1:** Human resources for health and population involved in the implementation of the vector surveillance-response system in five sectors, municipality of Totogalpa

Sector	VCT	PHC Staff	Communities	Houses	Population
1	1^a^	7	11	778	4 522
2		2	4	191	1 053
3		3	6	275	1 688
4		3	8	480	2 486
5		3	10	579	3 573
Total	1	18	39	2 303	13 322

Source: Ministry of Health in Nicaragua (2012)

*VCT* vector control technician, *PHC* primary health care

^a^There is one VCT at the municipal-level health centre

### Milestones of vector control

In Totogalpa, several vector control activities had been carried out since 1999 to control Chagas disease. As *R. prolixus* was found in the first national entomological survey in 1999, the MoH conducted a large-scale spraying programme in the municipality. As a result, *R. prolixus* has not been found since 2001. JICA’s technical cooperation started in 2009 and an entomological survey in 2010 investigated 360 randomly-selected houses, of which 11 (3.1%) were found to be infested with *T. dimidiata* (MoH, unpublished data). The proportion of infested houses was considered relatively low, so the MoH/JICA project proposed to introduce a vector surveillance-response system to maintain the low infestation level. The MoH and JICA started to introduce the initial model in 2012. Drawing on experience of the first year of operation, the vector surveillance-response system was redesigned in 2013. The municipality of Totogalpa also received financial aid from PAHO/WHO to conduct a large-scale spraying programme against *T. dimidiata* and treated more than 2 000 houses in 37 communities between November 2013 to February 2014. In 2014, the JICA ended its technical cooperation project and the vector surveillance-response system was expected to be run by the local health systems without external support.

### System design

We used a participatory and learning-by-doing approach to design and adjust the vector surveillance-response system in Totogalpa. Considering the high re-infestation capacity of *T. dimidiata* and limited local resources, we agreed that the surveillance-response system had to be effective, feasible and sustainable. We held several workshops to discuss the basic system design, inviting all potential stakeholders, such as departmental and municipal health authorities, the VCT, PHC teams and community health volunteers.

**Fig. 2:**
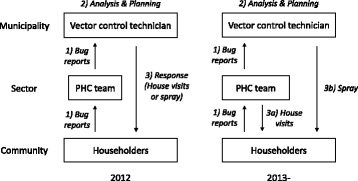
Two models of the vector surveillance-response system implemented in the municipality of Totogalpa, Nicaragua

The initial model is illustrated on the left-hand side of Fig. 2, showing that 1) community members would capture and report bugs to the nearest health centre or health post, 2) the VCT would collect and analyse the data to plan a response, and then 3) provide house visit or spraying as response. This model was implemented in January 2012 and we monitored its performance for a year. Having learnt from the first year of pilot experience, the system design was reviewed and revised, involving all stakeholders. In the revised model (Fig. 2, right), the task of responding is shared between the PHC teams, who became responsible for house visits (3a) and the VCT, who retained responsibility for spraying (3b). In this revised model, the PHC teams could decide on a house visit without waiting for data analysis and planning by the VCT. Community health volunteers could also conduct house visits or spraying after training and coordination.

In both models, visits were expected to be made to any house that reported vectors. During the house visits, the visiting personnel were expected to conduct a rapid house inspection and provide recommendations to help householders prevent vector infestation. For spraying, we established criteria using the percentage of houses reportedly infested in a given community during a given 6-month period (January–June or July–December). When more than 20% of houses reported vectors, all houses in that community were targeted for spraying. When the percentage was more than 5% and under 20%, only houses reporting infestations were to be sprayed. If the percentage was under 5%, the community was excluded from the spraying. The sprayers used residual insecticide, in line with national guidelines for indoor spraying techniques.

### Implementation

We prepared operational guidelines for each model, which were distributed to departmental and municipal health authorities. The guidelines were used to provide training and guidance to all staff and volunteers involved (i.e., the VCT, PHC teams and community health volunteers). Then, the PHC teams and community health volunteers encouraged householders to report vectors, utilizing various opportunities such as community meetings, outreach activities, waiting room at health facilities, radio campaign, and so on.

The performance of the vector surveillance-response system was evaluated semi-annually by national, departmental and municipal health authorities and the JICA project staff. This semi-annual evaluation was continued until 2014, when the JICA’s technical cooperation project ended. To assess financial feasibility, the JICA project decided not to provide any of the running costs of the vector surveillance-response system, including salaries, travelling costs, procurement of insecticide or office supplies. The JICA’s funding was limited to indirect support, such as reproduction of guidelines and organization of workshops and training. The cost of the semi-annual evaluation meetings was shared between the JICA project and the MoH.

### Data collection and analysis

We collected data from January 2012 to December 2015. At the Totogalpa Health Center, a VCT was responsible for registering vector reports and responses in a digital database. To assure the data quality, he double-checked the digital database monthly against the manual register maintained by the PHC teams. Each vector report included the name and address of the householder reporting the vectors, the date of vector capture, the date of vector receipt at the Health Center, vector species, number of reported vectors by developmental stage (nymph or adult), site of vector capture (intra- or peridomestic area), date of house visits, date of spraying, and profession of the person who responded. Among these data items, the name and address of the householder and the date and site of vector capture were provided by householders and the others were generated by health personnel.

To evaluate the performance of the vector surveillance-response system, we used three indicators, namely, amount of vector reports, response rates and response timeliness. First, we analysed the amount of vector reports by time and space. Quarterly trends of vector reports were visualized by drawing a line graph. Geographical clusters, where the vector reports were likely to be more frequent, were identified by retrospective space–time scan analysis with a Poisson model using SaTScan version 9.4.2 software. The outputs of the space–time scan analysis were visualized using QGIS version 2.12.0 software. Second, to assess the response rates, we defined response rates as number of houses where actions were taken divided by number of houses eligible for responses. They were computed for each 6-month period and separately for each type of response using the following formulae: 1) response rate for house visits = number of houses visited/number of houses reportedly infested × 100, and 2) response rate for spraying = number of houses sprayed/number of selected houses for spraying × 100. Third, we also evaluated timeliness of house visits. Timeliness was defined as days between vector capture and house visit. We visualized timeliness by using a beeswarm plot. To compare the timeliness by year and profession of service providers, we applied nonparametric statistical tests at a significance level of 5%. All graphs were created using EZR version 1.32 software and the statistical tests were processed using STATA version 13.1 software.

## Results

The vector surveillance-response system ran continuously in the municipality of Totogalpa throughout the study period, from January 2012 to December 2015.

### Trends of vector reports

**Fig. 3:**
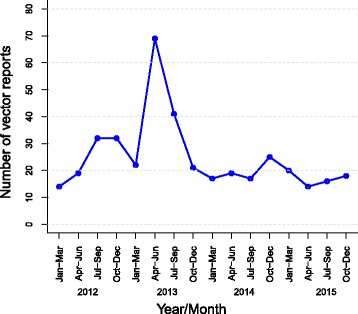
Quarterly trends in vector reports for *Triatoma dimidiata*, Totogalpa, 2012–2015

During the study period, a total of 396 reports were generated for *T. dimidiata*, with an average of 8.3 reports per month. Figure 3 shows quarterly trends in vector reports by householders. After the vector surveillance-response system started in 2012, the number of vector reports by householders rose to a strong peak in the second quarter of 2013. Similar peaks were not observed in other periods. From 2014 to 2015, vectors were reported on a relatively steady basis with an average of 6.1 reports per month. No seasonality was observed.

**Fig. 4:**
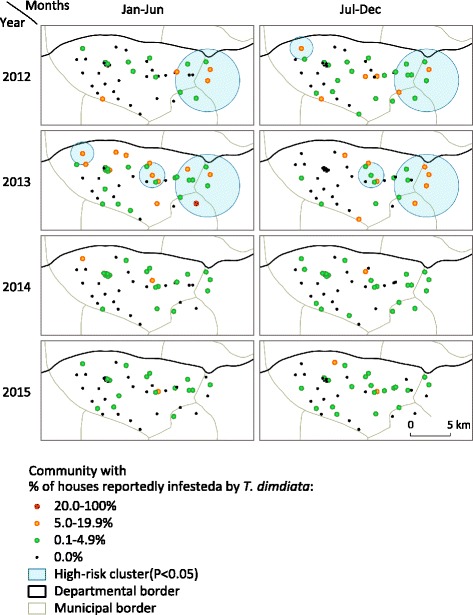
Spatiotemporal trends in vector reports by householders, Totogalpa, 2012–2015

Figure 4 shows the spatiotemporal trends in vector reports, identified by mapping the proportion of houses reportedly infested by *T. dimidiata* for each community in every 6-month period. Of the 39 communities in Totogalpa, only one community was found with more than 20% of houses infested in the first half of 2013 (red dot). In every 6-month period, the vector surveillance-response system found at least one community with between 5.0% and 19.9% of houses infested (orange dots). According to the response criteria, these communities (red or orange) were targeted for insecticide spraying. The communities reporting *T. dimidiata* in less than 5% of houses (green dots) were widely distributed throughout the study period and area. The space–time scan identified three clusters with a significantly higher likelihood of reporting infested houses (Fig. 4). The most likely cluster was in the eastern part of Totogalpa, and consisted of nine communities from 2012 to 2013. Within this cluster, the relative risk of reporting infested houses was 3.8 times higher than elsewhere (*P* < 0.001). After 2014, no clusters were seen, although *T. dimidiata* were reported continuously at a low level.

In the study period, a total of 570 *T. dimidiata* specimens were reported, of which 392 (69%) were adult insects found in intradomestic areas, 110 (19%) were nymphs from intradomestic areas and 68 (12%) specimens were found in peridomestic areas. No *R. prolixus* were reported.

### Response rates

Response rates in the vector surveillance-response system varied widely by time and response types. Table 2 summarizes the performance of the vector surveillance-response system in every 6-month period. The rate of house visits started at around 90% in 2012 and reached 100% in 2013. In 2014, the response rate decreased to 63% in the first half of the year, but recovered to 100% in the next period. However, in 2015, we observed a rapid decline to below 40%. In every 6-month period, the vector surveillance-response system identified eligible houses for spraying, using the established criteria. The response rate reached 100% in the second half of 2013, but declined rapidly to zero through 2014 and 2015.

**Table 2:** Response rates of the vector surveillance-response system, Totogalpa, 2012–2015

Type of response	2012		2013		2014		2015	
	Jan-Jun	Jul-Dec	Jan-Jun	Jul-Dec	Jan-Jun	Jul-Dec	Jan-Jun	Jul-Dec
House visit								
Houses with bug reports	29	56	82	55	35	33	27	28
Houses visited	26	53	82	55	22	33	18	11
Response rate (%)	89.7%	94.6%	100.0%	100.0%	62.9%	100.0%	66.7%	39.3%
Insecticide spraying								
Houses eligible for spraying	15	24	73	37	7	3	4	5
Houses sprayed	0	1	58	37	0	0	0	0
Response rate (%)	0.0%	4.2%	79.5%	100.0%	0.0%	0.0%	0.0%	0.0%

**Fig. 5:**
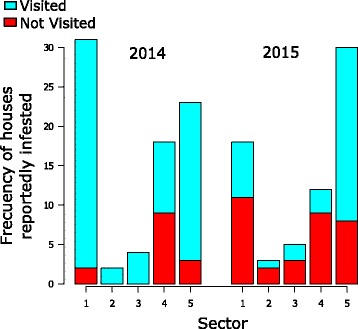
Houses with reported infestations visited (blue) and not visited (red) in the five sectors of Totogalpa, 2014–2015

The response rates for house visits did not vary among the five sectors in 2012 and 2013, when more than 90% of reportedly infested houses were visited. In these 2 years, it was rare not to visit an infested house. However, in the next 2 years, the rate of house visits became different across the five sectors. Figure 5 shows the distribution of visited and not visited infested houses at sector level during 2014 and 2015. In 2014, the sector with most infested houses not visited was Sector 4, where half of reportedly infested houses were not visited. In the same period, Sectors 1 and 5 also failed to visit some houses, but their response rates remained over 85%. In 2015, the response rates declined in all sectors. In four sectors, the response rates did not reach 40%, with Sector 4 worst at 25%. Only Sector 5 maintained a relatively high response rate of 73%. We observed high variability in rates of house visits across the five sectors in 2014 and 2015.

### Timeliness of responses

**Fig. 6:**
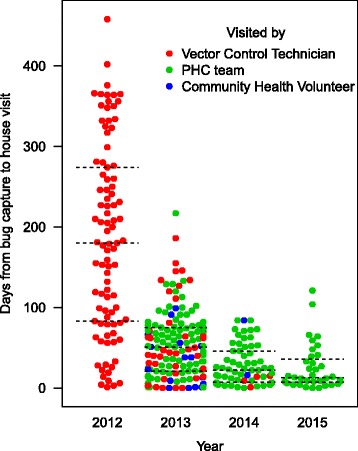
Timeliness of house visits by health personnel responding to vector reports, Totogalpa, 2012–2015. The horizontal dotted lines indicate quartiles

Timeliness of response improved throughout the 4-year study period. Figure 6 shows time in days between vector capture by householders and house visits by health personnel. In the first year of the pilot study, house visits were often delayed. The median time lag between bug capture and house visit was 180 days in 2012. The response time reduced after we modified the model of the surveillance-response systems at the beginning of 2013. The median time lag decreased significantly to 51 days in 2013, compared to 2012 (Wilcoxon rank-sum test, Z = 8.98, *P* < 0.001). Timeliness of providing house visits further improved as the median time lag was 22 days in 2014 and 13 days in 2015 (Fig. 6).

The improved timeliness was observed after the responsibility for house visits moved from the VCT to PHC staff. In 2012, all house visits were conducted by the VCT, but more than 70% of them were conducted by the PHC staff during 2013–2015 (Fig. 6). The median time lag between bug capture and house visit was 117, 31.5 and 23 days for the VCT, PHC staff and community health volunteers, respectively. A Kruskal–Wallis H-test shows that these differences were statistically significant (*χ* ^2^(2) = 69.405, *P* < 0.001). Figure 6 also shows that the variability of time lag between vector capture and house visits also reduced after modifying the system design. The interquartile range decreased from 191 days in 2012 to 54.5 days in 2013. The system’s responsiveness, in terms of timeliness, was largely improved after we modified the system design.

## Discussion

In this 4-year pilot study, we demonstrated a workable model for an entomological surveillance-response system for Chagas disease control in Nicaragua. The system was administrated within the resource-constrained settings, even after the end of the external technical cooperation project. Despite some challenges and limitations, we argue that the proposed vector surveillance-response system would be functional in Nicaraguan settings. In this section, we will first discuss the performance of the proposed vector surveillance-response system in Nicaragua. Then we argue lessons and implications of our study in broader settings, followed by this study’s limitations.

### Performance of the vector surveillance-response system in Nicaragua

Our study provided an evidence that the community residents kept reporting vectors during the study period, but the temporal trend of vector reports showed a wide range of fluctuation (Fig. 3). This inconstant trend of vector reports suggests three underlying scenarios in our study. First, the gradual increase of vector reports during the first year suggests that it took time to disseminate guidance and encourage community residents to report vectors. Many of the community residents in Totogalpa may not have been aware of the importance of reporting vectors in 2012. Second, the sudden increase of vector reports in the second quarter of 2013 may demonstrate an effect of seasonality. Such seasonality in reports of *T. dimidiata* has been described previously in Belize [30] and Mexico [24, 31], where non-domiciliated *T. dimidiata* is prevalent. However, it remains unclear whether there was any seasonality in house infestation of *T. dimidiata* in our study site because there were no seasonal trends in vector reports in other years. Third, the large-scale insecticide spraying conducted from November 2013 to February 2014 seems to have reduced the domestic population of *T. dimidiata* significantly, which led to low levels of vector reports in 2014 and 2015. This scenario is supported by the space–time analysis, which did not identify any high-risk cluster after the large-scale insecticide spraying (Fig. 4). In our study, the trends in vector reports are likely to be shaped by the system’s implementation process, vector ecology and public health intervention.

Responses to vector reports were provided by health personnel throughout the study period, but the response rates varied widely by time and type of response. The rate of house visits ranged from 60% to 100%. Figure 5 suggests the failure of house visits began in Sector 4 in 2014 and became systematized at the municipal level in 2015. Sector-level obstacles may include frequent turnover of PHC staff. To fill gaps in human resources, the MoH often sends medical students to rural areas as PHC team leaders. These medical students are transferred after 1–2 years of duty training in rural areas. The frequent rotation of PHC staff, with insufficient on-the-job training, may affect the response to vector reports. At the municipal level, factors contributing to the lower response rates could include weak leadership and lower levels of monitoring. Leadership is one of the crucial aspects in sustaining a disease control programme within the wider health system [32]. Analysis of similar vector control efforts for Chagas disease in Guatemala, El Salvador and Honduras found the responsiveness of the vector surveillance-response system was significantly associated with monitoring by higher-level health offices [26].

An outbreak of chikungunya virus in 2015 could be another reason to explain the low rates of house visits in the last year of our pilot study. Chikungunya was first reported in Nicaragua at the end of 2014 and the MoH was urged to intensify mosquito control and early case detection for this new infection. This newly-prioritized problem increased the workload for many healthcare workers, including the VCT and PHC staff, making difficult to provide house visits for Chagas disease control. In 2016, Central American countries also face the threat of Zika virus, which is transmitted by the same mosquito that carries dengue and chikungunya. Since Zika virus is expected to spread in the same way as chikungunya [33], the lower levels of house visits for Chagas disease will probably continue. If no action is taken by health authorities, the vector surveillance-response system may even stop spontaneously. Communicable disease control programmes need to be flexible and adaptable to external pressures if they are to be sustainable [32]. Ongoing cycles of reflection, planning and action are needed to make programmes sustainable [34].

The responses rate of insecticide spraying tended to be zero, except in 2013. High rates of 80-100% in 2013 must be attributed largely to the financial opportunity provided by PAHO/WHO to conduct spraying activities in Totogalpa. The external funding would play a crucial role not only to cover operational expenses but also to prioritize the spraying activities among other health services. The low response rates in general imply there would be political and logistical difficulties in organizing the spraying activities under the local health system.

Several challenges persist to sustaining the vector surveillance-response system in Nicaragua. First, the system must be made more resilient to internal changes such as frequent transfer of health personnel, and external pressures involving outbreak of other infectious diseases. Sustained disease control demands ongoing maintenance of capacity and long-term political commitment [35]. Leadership is needed to keep adapting the vector surveillance-response system. Second, the response package should be reviewed to improve the effectiveness of control of the domestic vector population. Our pilot study shows that there were limitations to the provision of insecticide spraying. The response package should therefore be revised to provide more feasible and effective interventions. For example, a positive deviance study in Ecuador identified that family members in triatomine-free houses performed habitual maintenance of the house, fumigation of dwellings, sweeping with insect-repellent plants and relocation of domestic animals away from the house [36]. These simple practices may be useful in Nicaragua, and health personnel could provide support to family members to find and disseminate locally-available anti-triatomine practices during the house visits. This approach, including community mobilization and empowerment, would be key to sustainable vector control for Chagas disease [37].

Third, as *T. dimidiata* is distributed widely in Nicaragua, it is necessary to scale up the vector surveillance-response system to national level. The Nicaraguan MoH set out national plans to regulate Chagas disease control in the country, including national implementation of the vector surveillance-response system [38]. These plans are expected to be introduced, with particular attention on establishing a mechanism for monitoring and supervision at national and departmental levels. Fourth, more scientific evidence is necessary to assess the entomological impact of the sustained vector surveillance-response system. Further research should involve regular entomological house inspection to see if the system contributes to suppressing the domestic vector population.

### Lessons and implications from the pilot study

One of the important functions of the surveillance-response system is dynamic mapping of transmission [3]. The vector surveillance-response system that we implemented in Nicaragua is capable of mapping transmission risk on a regular basis (Fig. 4). Since our system collects entomological data, it does not directly provide a picture of ongoing disease transmission. However, as approximately 10–30% of *T. dimidiata* specimens are infected with *T. cruzi* in Nicaragua (MoH in Nicaragua, unpublished data), the entomological mapping could be used to identify high-risk transmission areas. These data should, however, be interpreted carefully because passive surveillance tends to underestimate the true vector infestation level, because of underreporting by community residents. High-risk clusters can be identified using the passive surveillance data when the true vector infestation level is high and the community residents continue to report vectors. In contrast, the data should never be used to determine low-risk areas because the fewer vector reports do not necessarily mean that the true vector infestation level is low. Similarly, the data are not useful to estimate house infestation specifically by either peridomestic or nymph-stage vector population, because these bugs are less visible to householders. In spite of these data characteristics, our pilot study shows that the vector surveillance-response system can provide useful entomological information at low cost. Although the conventional three-phase vector control system proposes that vector surveillance should follow large-scale insecticide spraying, we recommend implementing community-based vector surveillance first, even in areas where the attack phase has not been carried out.

Our pilot study shows how the performance of the vector surveillance-response system may depend on wider health system issues. Experts recommend integrating NTD control with national PHC systems to assure long-term and sustainable programmes [39]. Understanding the interaction between disease-specific programmes and the broader health system is crucial for developing a sustainable control strategy [32]. In our pilot study, a timely response was considered important to satisfy householders, whose reporting behaviour is key to a sustainable vector surveillance-response system. The unacceptable response delays in the first year therefore required us to involve the PHC teams for better response timeliness. When we made the PHC teams responsible for house visits, we saw that some staff were reluctant to take on this responsibility, perhaps because they were generally overworked. We suggested that the PHC teams should not visit single houses, but instead maximize the use of their routinized outreach activities. Then the PHC teams started to include house visits for Chagas disease control as part of their regular work round. Integrating the vector surveillance-response system into the PHC system required clear role definition and improved managerial capacity. The participatory and learning-by-doing approach was a key contributor to changing the model, so that changes fitted the local health system and particularly its capacity.

In Latin America, the health sector reforms since 1990s have weakened communicable disease control programmes, because decentralization led to the conversion of vertical programmes into horizontal ones without careful consideration of local capacity [40]. Vector control programmes, in particular, became fragmented, a lower priority, and often run by untrained staff, making them ineffective [41, 42]. Our study in Nicaragua provides an example of reorganizing vector control programmes under the current trends in Latin American health systems. In Honduras, a similar vector control programme for Chagas disease could be integrated into PHC services, when health centres have managerial capacity [27]. Currently, some Latin American countries are in the process of reviewing the structure of PHC, in line with PAHO/WHO recommendations [28]. For example, in El Salvador, the MoH has introduced a new PHC model called Family Health Community Teams [43], similar to the model in Nicaragua. Our experience should inform other countries needing to integrate vector control programmes into new PHC systems. Such programme integration provides opportunities not only to increase the sustainability of disease-specific control programmes [32] but also to strengthen the broader health systems. Atun et al. [44] argue that programme integration improved both tuberculosis control programmes and the strength of the overall health system. We believe that the integration of the vector surveillance-response system for Chagas disease with PHC services creates an opportunity to strengthen the broader healthcare system by improving the leadership and managerial capacity of the PHC service providers.

Our pilot study casts doubt on the possibility of eliminating Chagas disease transmission by *T. dimidiata* in Central America. At least, it is evident that our vector surveillance-response system is not able to eliminate the risk of vector-borne transmission from the study area. This is because, first, it cannot detect unreported vectors. If householders do not report vectors to health personnel, the risk of disease transmission remains unnoticed. Second, even when the householders report vectors, the surveillance-response system cannot avoid a certain amount of delay in response. *T. cruzi* could be transmitted to family members during this delay. Third, and most importantly, insecticide was applied in a very limited number of houses. The spraying criteria set out that houses should be sprayed only when more than 5% of houses are reported to be infested in a community during a 6-month period. Even where these criteria were met, the selected houses were not sprayed in 2014 and 2015. Figure 4 shows that *T. dimidiata* were continuously reported over a widespread area, albeit at low levels, despite all the vector control works done in this municipality. This suggests that ongoing vector-borne disease transmission must be assumed. It seems unlikely that the vector surveillance-response system can eliminate vector-borne Chagas disease transmission in the near future. The vector surveillance-response system should therefore be viewed as a way to reduce disease transmission on an ongoing basis. This confirms the argument that an international evaluation scheme for the control of native species must be revised, as the existing scheme asserts that vector-borne Chagas disease transmission could be eliminated [22]. Our findings suggest that elimination of *T. cruzi* transmission by *T. dimidiata* is not achievable in reality.

### Limitations

Our study had two main limitations. First, we used secondary data generated by the MoH. These data might contain some errors such as missing information or underreported activities. Second, our geographical variability is limited. Our results come from a pilot study in just one municipality. Our findings may therefore not be directly applicable to other settings. However, despite these limitations, this study is the first longitudinal and in-depth study to assess Nicaragua’s ongoing vector surveillance-response system for Chagas disease. We believe this study provides considerable insights not only for Chagas disease control but also for implementing sustainable control programmes for other NTDs.

## Conclusions

This study demonstrates an example of a workable vector surveillance-response system for Chagas disease in a resource-constrained setting. The entomological surveillance through vector reports by householders was useful and sustainable, but the response to vector reports should be carefully monitored and improved. Key to sustaining the vector surveillance-response system is integration with PHC services, which in this study improved the timeliness of responses to reports. We suggest that the vector surveillance-response system should be maintained to monitor and suppress vector infestation of houses, although our data imply that elimination of vector-borne Chagas disease transmission is not feasible in Nicaragua. Continual improvement to make the vector surveillance-response system more resilient would create sound opportunities to strengthen the broader health system in the country. We believe that the findings from this study provide lessons for the control of Chagas disease and other NTDs requiring sustainable surveillance-response systems in resource-constrained settings.

## Additional file

Additional file 1: Multilingual abstracts in the six official working languages of the United Nations. (PDF 740 kb)

## Abbreviations

JICA: Japan International Cooperation Agency
MoH: Ministry of health
MOSAFC: Modelo de Salud Familiar y Comunitario (Family and Community Health Model)
NTDs: Neglected tropical diseases
PAHO: Pan American Health Organization
PHC: Primary health care
VCT: Vector control technician
WHO: World Health Organization.
