# Supplementary material for: Implementing a vector surveillance-response system for chagas disease control: a 4-year field trial in Nicaragua
Source: Infect Dis Poverty. 2017 Mar 6;6:18. doi: 10.1186/s40249-016-0225-7 (PMC5338093; doi:10.1186/s40249-016-0225-7)
Supplement: Additional file 1: — Multilingual abstracts in the six official working languages of the United Nations. (PDF 740 kb) [file 40249_2016_225_MOESM1_ESM.pdf]

Translation of the abstract into the six official working languages of the United Nations

تنفيذ نظام للملاحظة والاستجابة لناقل مرض شاغاس: تجربة ميدانية لأربع سنوات في نيكاراغوا

كوتا يوشيوكا، ودوريبل ترسيرو، وبيرون تيريز، وجيروا نكامورا، ولينين بيريز

ملخّص:

معلومات أساسية: مرض شاغاس هو أحد الأمراض المداريّة المُهْمَلة. وتشمل الجهود الدولية للسيطرة عليه في القضاء على أسلوب نقل المرض. وتواجه بلدان أمريكا الوسطى تحديات في إنشاء برامج مستدامة للسيطرة على ناقل للمرض، نظراً لأنه لا يمكن القضاء على الناقل الرئيس للمرض (ترياتوما ديميدياتا). في عام 2012م، بدأت وزارة الصحة في نيكاراغوا اختبارات ميدانية لنظام للملاحظة والاستجابة لناقل المرض، وذلك للسيطرة على تفشّي المرض. وتُقدّم هذه الورقة تقريراً عن النتائج الرئيسة لتلك الدراسة التجريبية.

الطُرُق: أُجريت هذه الدراسة في الفترة بين عاميْ 2012 و2015، في بلدية توتوقالبا. وقدمت الوكالة اليابانية للتعاون الدولي دعماً فنياً لتصميم ومراقبة نظام الملاحظة والاستجابة حتى عام 2014م. وشمل النظام: 1) تقارير عن الأُسَر إلى المرافق الصحية بشأن ناقل المرض؛ 2) تحليل البيانات والتخطيط لحالات الاستجابة في المراكز الصحية التابعة للبلدية؛ 3) زيارات للمنازل، أو رش المبيدات بواسطة عمال الصحة على سبيل الاستجابة. وقد سجّلنا جميع تقارير ناقل المرض وحالات الاستجابة في قاعدة بيانات الكترونية. وأُستخدمت البيانات التي جُمعت في وصف وتحليل أداء النظام، من حيث حجم تقارير ناقل المرض، وكذلك معدلات، وتوقيت حالات الاستجابة.

النتائج: سُجّلت 396 حالة لمرض ترياتوما ديميدياتا خلال مدة الدراسة. وحدّد التحليل الزماني والمكاني بعض المجموعات ذات المخاطر المرتفعة. وزار المسؤولون الصحيّون في عام 2013 جميع المنازل الموبوءة بالمرض، وانخفض معدل الاستجابة إلى 39٪ في عام 2015م. كما ازدادت معدلات رش المبيدات إلى أكثر من 80٪ في عام 2013م، لكن لم تُنفّذ أعمال الرش في العامين التاليين. وكان توقيت الزيارات المنزلية قد تحسّن بصورة ملحوظة بعد أن آلت المسؤولية من فنّي لمراقبة ناقل المرض، إلى مسؤولي الرعاية الصحية الأولية.

الخلاصة: نعتقد أن نظام الملاحظة والاستجابة المقترح لناقل المرض عمليّ في ظل محدودية موارد النظام الصحي في نيكاراغوا. كما أن الاندماج مع نظام خدمات الرعاية الصحية الأولية مسألة أساسية لتحسين أداء النظام. وتُعدّ الجهود المستمرة ضرورية لتحقيق التوافق بين نظام الملاحظة والاستجابة، والنظام الصحي الفعّال. وناقشنا أيضاً أن القضاء على ناقل المرض يظل هدفاً بعيد المنال. وتقدّم هذه الورقة دروس ليس عن السيطرة على مرض شاغاس في أمريكا الوسطى فحسب، بل لجميع جهود السيطرة للأمراض المدارية المهملة الأخرى التي تحتاج لنظام مستدام للملاحظة والاستجابة لدعم القضاء على المرض.

Translated from English version into Arabic by Hassan Adam, through

TRANSLATORS
WITHOUT BORDERS

美洲锥虫病的媒介监控响应系统的实施：在尼加拉瓜的 4 年试点

Kota Yoshioka, Doribel Tercero, Byron Pérez, Jiro Nakamura, Lenin Perez

摘要

引言：美洲锥虫病是一种被忽视的热带病。该病的国际防控目标包括消灭媒介传播。由于它的主要媒介是二分锥蝽（*Triatoma dimidiata*）不能被消灭，所以在建立可持续媒介控制方面中美洲国家面临着严峻挑战。 在 2012 年，尼加拉瓜卫生部开展了媒介监控响应系统以控制当地媒介的侵染。本文主要报告了这项试点研究的主要结果。

方法：本研究于 2012-2015 年在尼加拉瓜 Totogalpa 市开展。2014 年之前，日本国际合作机构为

媒介监测-响应系统提供设计和监测方面的技术支持。该系统包括：1）由住户提供给卫生设施的媒介报告；2）市卫生中心的数据分析与响应规划；3）卫生人员上门探访或喷洒杀虫剂。我们在数字数据库中录入以上所有媒介报告和响应工作。所收集的数据用来描述和分析系统在媒介报告，以及响应率和及时性等方面的系统性能。

结果：在研究期间，二分锥蝽共报告 396 次。时空分析确定了一些高风险集群。2013 年所有探访的房屋均发现该媒介，2015 年该侵袭率将至 39%。2013 年房屋杀虫剂喷洒率超过 80%，但在接下来的两年中未进行杀虫剂喷洒。将上门探访人员的责任由媒介控制技术人员转移到初级卫生保健人员以后，上门探访的及时性显著提高。

结论：我们认为该媒介监测-响应系统在资源有限的尼加拉瓜卫生系统内是具有可行性的，并且将其整合至初级卫生保健服务中是提高系统性能的关键。需要持续不断地努力使监控响应系统适应动态卫生系统。同时，我们认为消除媒介传播的目标仍无法实现。本文提供的经验不仅适用于中美洲地区的美洲锥虫病控制，而且也为需要可持续的监测-响应系统的其他被忽视热带病的防控提供经验。

Translated from English version into Chinese by Yin-Long Li, edited by Pin Yang

Mise en œuvre d'un système de surveillance et de réponse/réactions des vecteurs pour maîtriser la maladie de Chagas, un essai de quatre ans sur le terrain au Nicaragua

Kota Yoshioka, Doribel Tercero, Byron Pérez, Jiro Nakamura, Lenin Perez

Résumé

Historique: La maladie de Chagas est une des maladies tropicales laissée à l'abandon (NTDs). Pour la maîtriser, les objectifs internationaux nécessitent l'éradication (de la maladie) à transmission vectorielle. Les pays de l'Amérique Centrale font face à des défis en élaborant des méthodes de lutte anti-vectorielle durables, puisque le vecteur principal *Triatoma dimidiata*, ne peut pas être éliminé. En 2012, le ministère de la santé au Nicaragua a commencé un essai sur le terrain d'un système de surveillance et de réaction des vecteurs aux fins de maitriser l'invasion du vecteur domestique. Ce document rapporte les principales conclusions de cette étude pilote.

Méthodes: Cette étude a été effectuée de 2012 à 2015 dans la municipalité de Totogalpa. L'Agence de Coopération Internationale du Japon a apporté une coopération technique dans la conception et le suivi d'un système de surveillance et de réponse/réaction jusqu'en 2014. Cc système a intégré 1) des rapports vectoriels allant des ménages aux établissements de soins. 2) l'analyse des données et l'organisation des réponses/réactions au centre de santé municipal et 3) des visites à domicile ou des pulvérisations d'insecticide par le personnel de la santé à effet de réponse/réaction. Nous avons enregistré tous les rapports et les réponses/réactions dans une base de données numérique. Les données collectées ont été utilisées pour décrire et analyser la performance du système en termes de quantités des rapports vectoriels ainsi que les taux et la rapidité des réponses/réactions.

Résultats: Pendant toute la durée de l'étude, le vecteur, *T. dimidiata* a été signalé 396 fois ; l'analyse spatio-temporelle a identifié quelques groupes à haut risque. Tous les domiciles signalés comme étant infestés ont été visités par le personnel de santé en 2013 et ce taux de réaction/réponse est tombé à 39% en 2015. Les taux de pulvérisations d'insecticide ont dépassé les 80% en 2013 mais aucune

pulvérisation n’a été effectuée au cours des deux années suivantes. La rapidité des visites à domicile s’est améliorée de manière significative après que le transfert des compétences d’un technicien de lutte anti-vectorielle au personnel de soins de santé primaires ait eu lieu.
Conclusions: Nous soutenons que le système de surveillance et de réponse/réaction proposé est applicable dans un système de ressources limitées en matière de santé au Nicaragua. Intégrer les services de soins de santé primaire fut un élément fondamental pour améliorer la performance du système. Des efforts constants sont nécessaires pour continuer à adapter le système de surveillance et de réponse/réaction aux systèmes de santé dynamiques. Nous évoquons aussi que l’objectif d’éradiquer le facteur (de la maladie) à transmission vectorielle demeure irréalisable. Ce document tire des enseignements non seulement pour lutter contre la maladie de Chagas en Amérique Centrale, mais aussi pour les actions de lutte contre d’autres NTD’s qui nécessitent un système de surveillance et de réponse/réaction durable pour renforcer l’éradication.

Translated from English version into French by veromarie, through

TRANSLATORS
WITHOUT BORDERS

Внедрение системы реагирования на обнаружение переносчика в целях борьбы с болезнью Шагаса: 4 года применения на практике в Никарагуа

Kota Yoshioka, Doribel Tercero, Byron Pérez, Jiro Nakamura, Lenin Perez

Аннотация
Исходные данные: Болезнь Шагаса — одно из тропических заболеваний, которым не уделяют должного внимания (NTD). В задачи по борьбе с этим заболеванием на международном уровне входит, в числе прочего, исключение возможности передачи болезни переносчиками. Перед странами Центральной Америки стоит непростая задача создать жизнеспособную программу по борьбе с переносчиками, поскольку главного переносчика — триатомовых клопов *(Triatoma dimidiate)* — невозможно уничтожить полностью. В 2012 году в Никарагуа, по приказу Министерства здравоохранения, начались практические испытания системы реагирования на обнаружение переносчика, задачей которой была борьба с заражением от переносчика. В данной статье приводятся результаты этого экспериментального исследования.
Методы: Исследование проводилось с 2012 по 2015 гг. на территории муниципального образования Тотогальпа. Вплоть до 2014 года Японское агентство по международному сотрудничеству оказывало техническую поддержку в разработке системы реагирования и контроле её работы. Система заключалась в следующем: 1) домовладельцы сообщали медицинским учреждениям об обнаруженных переносчиках; 2) в муниципальном центре здравоохранения проводился анализ данных и составлялись планы по реагированию; 3) визит врача на дом или опрыскивание инсектицидом силами медицинского персонала. Все сообщения о переносчиках и принятые ответные меры были занесены в цифровую базу данных. Собранные данные были использованы для описания и анализа эффективности системы с точки зрения количества сообщений о переносчиках и масштаба и своевременности ответных мероприятий.

Результаты: В период исследования было получено 396 сообщений о *T. dimidiata*. Пространственно-временной анализ выявил районы высокого риска. В 2013 году медицинские работники посетили все дома, в которых, по сообщениям, были обнаружены триатомовые клещи, и количество таких посещений в 2015 году снизилось до 39%. Доля домов, в которых проводилось опрыскивание инсектицидом, в 2013 году превышала 80%, но в следующие два года не было произведено ни одного опрыскивания. Посещения специалистов стали значительно более своевременными после того, как ответственность была передана от специалиста по борьбе с переносчиками сотрудникам службы первичной медицинской помощи.
Выводы: Авторы считают, что предложенная система реагирования на обнаружение переносчиков работоспособна в рамках ограниченной в ресурсах системы здравоохранения Никарагуа. Интеграция в службу первичной медицинской помощи оказалась ключом к повышению эффективности работы системы. Адаптация системы реагирования на обнаружение переносчиков к постоянно изменяющейся системе здравоохранения невозможна без непрерывных усилий. Авторы также затрагивают вопрос о том, что цель полностью устранить передачу болезни переносчиками остаётся недостижимой. В статье не только приводится опыт борьбы с болезнью Шагаса в Центральной Америке, но и отстаивается необходимость бороться с другими NTD, что требует устойчивых систем реагирования на обнаружение переносчиков.

Translated from English version into Russian by Elena Alieva, through

TRANSLATORS WITHOUT BORDERS

Implementación de un sistema de respuesta y vigilancia del vector para el control de la enfermedad de Chagas: un estudio de 4 años en terreno en Nicaragua

Kota Yoshioka, Doribel Tercero, Byron Pérez, Jiro Nakamura, Lenin Perez

Resumen

Antecedentes: La enfermedad de Chagas es una de las enfermedades tropicales ignoradas (ETI). Los objetivos internacionales para este control implican la eliminación de la transmisión producida por un vector. Los países centro americanos enfrentan el desafío de establecer programas de control de vectores sustentables ya que el vector principal, *Triatoma dimidiata*, no puede ser eliminado. En el 2012, el ministerio de salud en Nicaragua comenzó una prueba en terreno de un sistema de respuesta y vigilancia de infestación de vector es doméstica. Este artículo informa sobre los hallazgos principales de este estudio piloto.
Métodos: Este estudio se realizó desde el 2012 hasta el 2015 en la municipalidad de Totogalpa. La Agencia de cooperación Internacional de Japón proporciono cooperación técnica para diseñar y controlar el sistema de vigilancia y respuesta hasta el 2014. Este sistema consistió en 1) informes de los habitantes sobre vectores a la los servicios de salud, 2) análisis de datos y planificación de una respuesta en el centro de salud municipal y 3) visitas domiciliarias o fumigación con insecticidas por el personal de salud como respuesta. Registramos todos los informes sobre vectores y respuestas en un banco de datos digital. Los datos recolectados se utilizaron para describir y analizar el rendimiento del sistema en relación a la cantidad de informes sobre vectores así como la frecuencia y respuestas

oportunas.
Resultados: Durante el periodo del estudio, *T. dimidiata* fue notificado 396 veces. Un análisis espaciotemporal identificó algunas agrupaciones de alto riesgo. Todas las viviendas que informaron estar infestadas fueron visitadas por personal de salud en el 2013 y esta velocidad de respuesta disminuyó a 39% en el 2015. La frecuencia de fumigaciones con insecticidas aumentó a más de 80% en el 2013 pero en los dos años siguientes no se realizaron fumigaciones. Las visitas a las viviendas en un tiempo oportuno mejoraron significativamente después que la responsabilidad fue trasladada del técnico de control del vector al personal de atención de salud primaria.
Conclusiones: Nosotros aducimos que el sistema propuesto de vigilancia y respuesta del vector es factible dentro del sistema de salud con recursos limitados de Nicaragua. La integración a los servicios de atención de salud primaria fue la clave para mejorar el rendimiento del sistema. Se necesitan renovados esfuerzos para seguir adaptando el sistema de vigilancia y respuesta a los dinámicos sistemas de salud. Asimismo comentamos que el objetivo de eliminar la transmisión producida por vectores, aún es inasequible. Este artículo proporciona lecciones no solo para el control de la enfermedad de Chagas sino también para los esfuerzos para controlar otras ETI que requieren sistemas de vigilancia y respuesta para promover su eliminación.

Translated from English version into Spanish by Claudia Guiraldes, through

TRANSLATORS
WITHOUT BORDERS
